# “Characterization of visual function parameters in relation to macular pigment optical density in a pediatric population”

**DOI:** 10.1007/s00417-025-06935-1

**Published:** 2025-09-08

**Authors:** Víctor Ponce-García, María-José Bautista-Llamas, Marta-C. García-Romera

**Affiliations:** Department of Physics of Condensed Matter, Optics Area. Vision Research Group (CIVIUS), University of Seville, Avenida de la Reina Mercedes s/n (41012), Seville, Spain

**Keywords:** Macular pigment optical density, Children, Visual function, Visual acuity, Refractive status, Contrast sensitivity, Lutein, Zeaxanthin

## Abstract

**Purpose:**

To analyze the relationship between various visual function parameters (refractive status, visual acuity and contrast sensitivity) and macular pigment optical density (MPOD) values, as well as dietary intake of lutein and zeaxanthin in a pediatric population.

**Methods:**

Thirty-six healthy White pediatric patients participated in this cross-sectional study conducted at the Optometry Clinic (Faculty of Pharmacy, Seville, Spain). MPOD values were measured using the MPSII^®^ (Macular Pigment Screener II). Visual acuity was assessed with the ETDRS (Early Treatment Diabetic Retinopathy Study) chart, and refractive status was determined through subjective refraction, recording sphere and cylinder for each eye. Contrast sensitivity was measured with Optotab+^®^ at key spatial frequencies under photopic conditions. Lutein and zeaxanthin intake was evaluated using the KIDMED questionnaire, categorizing children based on their adherence to the Mediterranean diet.

**Results:**

The mean age of participants was 7.67 ± 1.21 years. MPOD values were similar between the right eye (0.51 ± 0.16 du) and the left eye (0.47 ± 0.23 du), with no statistically significant difference observed (*p* = 0.51). Visual acuity in both eyes ranged from − 0.08 to 0.10 logMAR (logarithm of the minimum angle of resolution). Contrast sensitivity was assessed at spatial frequencies of 1.5, 3, 6, 12 and 18 cycles per degree for each eye. No significant interocular differences were found for any of the visual parameters evaluated. All children in the study had medium or high adherence to the Mediterranean diet; no participants met criteria for low adherence category. Additionally, no significant differences in visual function or MPOD were observed between children with medium or high adherence. No correlations were observed between MPOD and any ocular parameters in either eye.

**Conclusion:**

This study found no correlation between MPOD and visual function parameters in children with medium to high adherence to the Mediterranean diet. Furthermore, adherence to the Mediterranean diet – and the associated lutein and zeaxanthin intake – showed no significant association with MPOD values or visual performance in this pediatric sample. These findings suggest that children’s dietary habits may not influence MPOD values or visual function. Further studies with more diverse and larger pediatric populations, including children with low dietary adherence, are warranted to clarify these relationships.

**Supplementary Information:**

The online version contains supplementary material available at 10.1007/s00417-025-06935-1.

## Key message

*****What is known***:**

- Macular pigment optical density (MPOD) enhances visual performance in adults due to its antioxidant and light-absorption properties.
- MPOD may be considered a natural biomarker for cognitive and visual function.

*****What is new***:**

- In healthy White children with medium or high adherence to Mediterranean diet, no significant correlation was found between MPOD and visual acuity, refractive error, or contrast sensitivity.
- MPOD was not significantly associated with dietary intake of lutein and zeaxanthin rich foods, suggesting that other factors may influence its accumulation in children.

**Supplementary Information:**

The online version contains supplementary material available at 10.1007/s00417-025-06935-1.

## Introduction

Lutein (L) and Zeaxanthin (Z) are the two main xanthophyll carotenoids located in the macula lutea, the central region of the retina, designed to optimize visual function [1]. Since these macular pigments cannot be synthesized by human body, they must be ingested through dietary sources such as vegetables, fruits and eggs [2, 3]. The concentration of these macular pigments in retina is quantified as macular pigment optical density (MPOD), where higher MPOD values are associated with greater amount of L and Z [4]. Conversely, several factors have been associated with lower MPOD values, including inadequate dietary intake of macular carotenoids, obesity, excessive exposure to short-wavelength light, female gender, and smoking [5]. Nevertheless, these risk factors have been primarily studied in adult population. In children, genetic factors and even maternal dietary intake levels during pregnancy and breastfeeding appear to play crucial roles in determining MPOD values [6, 7].

The anti-inflammatory and antioxidant properties of L and Z have been observed as preventive factors for neurological diseases [2] and enhancing cognitive performance [8]. Furthermore, their antioxidant capacity avert to promote free radical formation and subsequently, oxidative damage in vulnerable tissues such as the retina. Besides, these carotenoids also absorb short-wavelength light, commonly referred to as blue light, thereby reducing the risk of retinal damage caused by oxidative stress and subsequent degeneration [2, 9]. This mechanism minimizes lipofuscin accumulation, which can lead to photochemical injury, drusen formation, as well as retinal pigment epithelium cell death [10]. With the increasing exposure to blue light exposition emitted by digital devices with LED lightning screens, which has been associated with myopia prevalence [11] and lower MPOD values [12], although this relationship remains unclear due to potential confounding factors such as age. This issue is therefore a growing concern.

The association between MPOD and visual performance has been widely explored in adult populations, with numerous studies reporting benefits in visual function parameters such as visual acuity (VA), glare disability, photostress recovery, chromatic contrast, and contrast sensitivity (CS) [13–16]. Nevertheless, the relationship between MPOD values and refractive status has not been thoroughly studied [17].

The vast majority of studies have been conducted in adult populations, and studies focusing on children remain scarce. Indeed, only four studies have examined MPOD in children in relation to visual functions such as spherical equivalent refraction and macular thickness, but none have provided data on CS, VA, or detailed refractive status [7]. Additionally, other studies have primarily investigated amblyopic populations rather than healthy children, or have not focused on correlations between optometric parameters and dietary intake patterns. Thus, it remains unclear whether visual function in children is significantly influenced by MPOD values.

This study aimed to (1) analyze the relationship between various visual function parameters not previously detailed in pediatric population, and (2) evaluate dietary intake in relation to MPOD values in children.

## Materials and methods

### Study protocol

This cross-sectional observational study enrolled 36 White children aged 6 to 9 years and was conducted at the Optometry Clinic of the Faculty of Pharmacy, University of Seville, Spain. The study was approved by the Ethics Committee of the University of Seville and adhered to the tenets of the Declaration of Helsinki. Informed consent was obtained from the participants’ parents prior their inclusion, and they were fully informed about the nature of the study. The ocular examinations were conducted by a trained optometrist, comprising a refractive examination with VA measurements, CS evaluation, slit-lamp biomicroscopy using the SL980 slit lamp (CSO, Italy) and MPOD analysis. Measurements were consistently obtained from the right eye first.

The inclusion criteria established were: (1) children capable of completing the MPOD task and (2) healthy White children. The exclusion criteria included: (1) evidence of any ocular pathology, such as lens opacity, corneal scarring, retinal disease or amblyopia; (2) VA worse than 0.1 logarithm of the minimum angle of resolution (logMAR) with best correction or a difference of more than two lines between eyes; (3) a history of systemic diseases or take any medication; (4) incomplete dietary questionnaire responses; and (5) inability to complete the MPOD task or unreliable results during MPOD analysis.

While the children were being examined, their parents were interviewed by another specialized optometrist regarding their children’s dietary habits using a standardized questionnaire, as described below.

### Macular Pigment Optical Density (MPOD) measurement

MPOD values were measured using MPS II^®^ (Macular Pigment Screener II) (Elektron Technology UK Ltd., Cambridge, UK), a macular densitometer employing the Heterochromatic Flicker Photometry technique. This method involves a central test stimulus alternating between two wavelengths: blue (460 nm), which is absorbed by macular carotenoids, and green (540 nm), which is not absorbed. The test presents a flickering disk, and participants press a button when they perceive flickering stimulus at different blue-green ratios. Upon completion, the device determines the measurements as reliable (green), partially reliable (yellow) or unreliable (red). MPOD values range from 0 to 1, expressed in density units (du).

### Visual function measures

Several ocular parameters were assessed in this study. Subjective refraction was performed using Essilor MPH100E S/N 000104 phoropter (Essilor, Paris, France), and spherical and cylindrical values were recorded in diopters (D) for each eye separately. Henceforth, the spherical equivalent refraction (SER) was calculated as the spherical values plus half of the cylindrical value. VA was explored using the Early Treatment Diabetic Retinopathy Study (ETDRS) chart nº 2110 (Precision Vision Inc., Illinois, USA) at a distance of 4 m under monocular conditions (right eye (RE) and left eye (LE)). Results were recorded in logMAR units with best-corrected refraction.

CS was evaluated using Optotab^®^+ (SmarThings4Vision, Zaragoza, Spain) under photopic conditions with best-corrected refraction. The assessed spatial frequencies included 1.5, 3, 6, 12 and 18 cycles per degree (cpd) for each eye separately. Participants were instructed to identify the orientation of each presented pattern (vertical, left, right or undetectable). At the end of the test, mean results for each spatial frequency was recorded in logarithmic units (log units).

### Dietary analysis

Lutein and zeaxanthin were assessed using the KIDMED questionnaire, developed by Serra-Majem et al. [18], which evaluates dietary patterns in children and adolescents. The questionnaire focuses on the consumption of fruits, vegetables, dairy products, fish and fats. Parents completed the 16 dichotomous (YES/NO) questions regarding their children’s diet. Each KIDMED questionnaire was scored, and adherence to the Mediterranean diet was categorized as follow: high (> 8 points), medium (4–7 points) and poor (≤ 3 points).

#### Statistical analysis

Statistical analysis was performed using SPSS© Statistic software version 26.0 for Windows (IBM Corporation, Armonk, NY, USA). Descriptive results are expressed as mean ± standard deviation. Data normality was assessed using Shapiro-Wilk test. Due to non-normal data distribution, the Wilcoxon test was used to compare differences between eyes. The Mann-Whitney U test was employed to determine differences between Mediterranean diet adherence categories and other variables. Spearman’s correlation coefficient was used to assess associations between MPOD values, visual function parameters, and KIDMED scores. A p-value (p) < 0.05 was considered statically significant, with a 95% confidence level.

## Results

The current study enrolled 36 children, including 30 boys and 6 girls with a mean age of 7.67 ± 0.21 years. Table 1 summarizes the demographic characteristics of the sample, assessed for each eye separately. When comparing MPOD values by sex, boys exhibited higher MPOD values than girls in both eyes. Nevertheless, the differences between genders were not statistically significant (*p* = 0.82 for RE and *p* > 0.99 for LE).

**Table 1:** Demographical characteristics

Mean ± SD		RE Mean ± SD	Min	Max	LE Mean ± SD	Min	Max
Sex (*n* = 36)							
MPOD Boys	30	0.50 ± 0.16	0.19	0.84	0.47 ± 0.22	0.05	0.86
MPOD Girls	6	0.49 ± 0.15	0.29	0.67	0.46 ± 0.32	0.14	0.82
Age	7.67 ± 1.21						
KIDMED score	7.58 ± 1.61						
MPOD value (du)		0.51 ± 0.16	0.19	0.84	0.47 ± 0.23	0.05	0.86
SER (D)		0.19 ± 0.88	−4.63	1.38	0.60 ± 1.52	−8.38	1.50
Sphere (D)		0.46 ± 0.80	−3.50	2.25	0.30 ± 1.46	−7.50	2.50
Cylinder (D)		−0.55 ± 0.45	−2.25	−0.25	−0.48 ± 0.46	−2.00	0.00
Visual acuity (logMAR)		0.06 ± 0.05	−0.08	0.10	0.07 ± 0.05	−0.08	0.10
Contrast Sensitivity 1.5 cpd (log units)		1.64 ± 0.15	1.23	1.94	1.63 ± 0.15	1.18	1.83
Contrast Sensitivity 3 cpd (log units)		1.96 ± 0.19	1.51	2.22	1.98 ± 0.15	1.65	2.22
Contrast Sensitivity 6 cpd (log units)		2.10 ± 0.15	1.57	2.22	2.11 ± 0.13	1.67	2.22
Contrast Sensitivity 12 cpd (log units)		1.97 ± 0.20	1.23	2.12	1.99 ± 0.14	1.48	2.12
Contrast Sensitivity 18 cpd (log units)		1.69 ± 0.23	0.85	2.23	1.71 ± 0.23	0.78	2.23

Abbreviations: cpd, cycles per degree; D, diopters; du, density unit; LE, left eye; log units, logarithmic units; MPOD, macular pigment optical density; RE, right eye; SER, spherical equivalent refraction. Values are shown as Mean ± standard deviation (SD)

When analyzing MPOD values for each eye, although a higher mean MPOD value was observed in the RE (0.51 ± 0.16 du) compared to the LE (0.47 ± 0.23 du), this difference was not statistically significant (*p* = 0.51).

Regarding visual function, all participants demonstrated good VA in both eyes, with values ranging from − 0.08 to 0.10 logMAR. The majority of children were hyperopic (≥ + 0.25 D) with a SER of 0.19 ± 0.88 D in RE and 0.60 ± 1.52 D in LE, with minimal astigmatism in both RE (−0.55 ± 0.45 D) and LE (−0.48 ± 0.46 D). Despite that, no significant differences were found between the eyes for VA, SER, sphere, or cylinder (*p* > 0.05).

In addition to refractive status, CS was evaluated at various spatial frequencies. Across all spatial frequencies, no significant differences were observed between both eyes (*p* > 0.05).

Regarding the dietary analysis, Table 2 categorizes the sample based on their adherence to the Mediterranean diet according to the KIDMED score. The mean score for the total sample was 7.58 ± 1.61 points. The vast majority of children (*n* = 20) demonstrated high Mediterranean diet adherence (8.78 ± 0.81 points), compared to those with medium adherence (6.13 ± 1.09 points).

**Table 2:** Factors divided depending on Mediterranean adherence

			Medium Mediterranean diet adherence (*n* = 16)	High Mediterranean diet adherence (*n* = 20)	*p*-value	
MPOD value (du)		Right eye	0.50 ± 0.16	0.50 ± 0.16	0.89	
		Left eye	0.46 ± 0.17	0.47 ± 0.28	0.80	
KIDMED score			6.13 ± 1.09	8.78 ± 0.81	**-**	
SER (D)		Right eye	0.37 ± 0.38	0.04 ± 1.13	0.26	
		Left eye	0.38 ± 0.65	−0.20 ± 1.94	0.12	
Sphere (D)		Right eye	0.67 ± 0.51	0.28 ± 1.00	0.17	
		Left eye	0.65 ± 0.84	−0.04 ± 1.88	0.16	
Cylinder (D)	Right eye		−0.59 ± 0.43	−0.54 ± 0.50	0.44	
	Left eye		−0.56 ± 0.57	−0.42 ± 0.37	0.65	
Visual acuity (logMAR)						
Right eye			0.06 ± 0.06	0.06 ± 0.05	0.91	
Left eye			0.06 ± 0.06	0.07 ± 0.04	0.72	
Contrast Sensitivity 1.5 cpd (log units)						
Right eye			1.67 ± 0.11	1.61 ± 0.18	0.38	
Left eye			1.65 ± 0.12	1.62 ± 0.18	0.60	
Contrast Sensitivity 3 cpd (log units)						
Right eye			2.02 ± 0.18	1.92 ± 0.18	0.05	
Left eye			2.00 ± 0.14	1.97 ± 0.16	0.65	
Contrast Sensitivity 6 cpd (log units)						
Right eye			2.11 ± 0.12	2.10 ± 0.18	0.58	
Left eye			2.12 ± 0.14	2.11 ± 0.14	0.67	
Contrast Sensitivity 12 cpd (log units)						
Right eye			1.98 ± 0.24	1.99 ± 0.14	0.32	
Left eye			1.97 ± 0.16	2.01 ± 0.13	0.54	
Contrast Sensitivity 18 cpd (log units)						
Right eye			1.66 ± 0.27	1.71 ± 0.21	0.46	
Left eye			1.67 ± 0.27	1.75 ± 0.21	0.96	

Abbreviations: cpd, cycles per degree; D, diopter; du, density unit; LE, left eye; log units, logarithmic unit; MPOD, macular pigment optical density; RE, right eye; SER, spherical equivalent refraction. Values are shown as Mean ± standard deviation (SD)

Note: The KIDMED score was used to categorize participants into medium and high Mediterranean diet adherence groups. Therefore, no statistical comparison was conducted for this variable, as the grouping was derived from it

When evaluating visual parameters, the medium Mediterranean diet adherence group exhibited more hyperopic values and higher astigmatic values in both RE and LE compared to the high adherence group. Additionally, the medium Mediterranean diet adherence group showed numerically higher CS values at lower spatial frequencies (1.5, 3 and 6 cpd), whereas the high adherence group exhibited slightly higher results at higher spatial frequencies (12 and 18 cpd). However, no statistically significant differences were found between the groups for any of the assessed visual parameters (*p* > 0.05), as shown in Table 2.

Spearman’s correlation coefficient was used to evaluate the relationship between MPOD values and visual function parameters. Tables 3 and 4 present the correlation results for RE and LE, respectively. No significant correlations were identified between MPOD values and any ocular characteristics for either eye.

**Table 3:** Spearman’s correlation coefficient between RE MPOD values and RE visual function parameters

	(ρ)	*p*-value
SER	−0.04	0.81
Sphere	−0.09	0.60
Cylinder	−0.07	0.70
Visual acuity	−0.24	0.16
1.5 cpd	0.10	0.56
3 cpd	−0.04	0.81
6 cpd	−0.09	0.57
12 cpd	0.04	0.79
18 cpd	−0.01	0.96

Abbreviations: cpd, cycles per degree; MPOD, macular pigment optical density; RE, right eye; SER, spherical equivalent refraction

ρ: Spearman’s correlation coefficient

*p-value < 0.05 is considered statistically significant

**Table 4:** Spearman’s correlation coefficient between LE MPOD values and LE visual function parameters

	(ρ)	*p *-value
SER	-0.29	0.09
Sphere	-0.32	0.06
Cylinder	0.08	0.63
Visual acuity	0.13	0.46
1.5 cpd	0.06	0.73
3 cpd	0.15	0.41
6 cpd	0.01	0.94
12 cpd	-0.04	0.80
18 cpd	-0.11	0.53

Abbreviations: cpd, cycles per degree; LE, left eye; MPOD, macular pigment optical density; SER, spherical equivalent refraction

ρ: Spearman’s correlation coefficient

*p-value < 0.05 is considered statistically significant

## Discussion

The present study aimed to investigate the relationship between central MPOD values in the retina and several visual parameters in healthy children, including VA, refractive status (SER, sphere and cylinder), and CS at key spatial frequencies. These parameters were assessed for each eye separately. Contrary to our initial hypothesis, no significant associations were found between MPOD and these visual parameters.

As opposed to studies conducted on adult populations, there is a lack of research analyzing MPOD values in relation to visual parameters in children. Additionally, the visual parameters evaluated in adult population often differ from those relevant to pediatric populations [7].

To our knowledge, this study is the first to analyze refractive status by separately sphere and cylinder values rather than using spherical equivalent refraction (SER) uniquely. This approach aimed to provide a more detailed characterization of MPOD and refractive status. Previous studies in healthy pediatric population reported only SER ranges, without specifying mean values or separating sphere and cylinder components [19, 20]. While these studies reported more myopic values, likely due to the inclusion of the cylindrical component (with tends to be more negative), our study found slightly hyperopic spherical values even when calculated as SER. Additionally, Garcia-Romera et al. [21] reported a refractive status prevalence and found lower MPOD values in myopic children. Despite these findings, our study did not identify any significant correlation between MPOD values and the visual parameters analyzed, which is consistent with previous studies. A larger sample size, with a wider range of refractive errors, may be required to detect such correlations, rather than analyzing emmetropic or low-to-moderate refractive errors. Furthermore, no previous studies have analyzed cylinder values in relation to MPOD.

Some studies have evaluated MPOD values and their correlations with visual parameters in amblyopic children by comparing the amblyopic eye to the fellow eye [22, 23]. Notably, the study conducted by Erkan et al. [22] included a group of healthy children and found significantly higher MPOD values in the preferred eyes of strabismic children compared to both non-preferred eyes and healthy eyes. In contrast, our study focuses exclusively on healthy children, ensuring a normative reference for comparison. Although Wang et al. [23] observed lower MPOD values due to macular deprivation, they reported no correlation between MPOD and SER. Furthermore, they did not find a relationship between MPOD and best-corrected visual acuity. Therefore, these results align with our study, demonstrating no significant association between MPOD and visual performance.

Concerning CS, no studies in children have specifically examined this parameter in relation to MPOD. CS has been predominantly assessed in adult populations, where correlations have been observed at 1º eccentricity and high spatial frequencies [13, 15]. In contrast, our study found no relationship between MPOD and any of the commonly evaluated spatial frequencies in children, suggesting that MPOD may not influence CS in this age group or that effect is too subtle to detect.

When children were categorized based on Mediterranean diet adherence, differences in KIDMED scores were observed, potentially reflecting variations in L and Z consumption. However, no significant differences were found between groups in any visual parameters assessed. Although this is the first study to explore visual correlations with MPOD based on Mediterranean diet adherence, other studies have reported higher MPOD values in children with medium adherence compared to those with poor or high adherence [21]. Consistent with our findings, these studies did not observe a correlation between KIDMED scores and MPOD values.

The lack of significant associations observed may suggest that other unmeasured factors have a stronger influence on MPOD during childhood such as genetic factors, [24] maternal diet during pregnancy, macular pigment deposition in the maternal uterus [25] or breastfeeding [26].

This study has several limitations. Firstly, the sample size could be increased by including a broader age range, particularly adolescents. Additionally, the gender distribution should be balanced to better explore potential sex-based differences. Moreover, incorporating a wider range of refractive errors would help determine whether MPOD values correlate with more pronounced refractive errors or CS variations. On the other hand, it is crucial to note that the use of the MPS II^®^ quantifies MPOD by providing a single value of MPOD, rather than generating a 2-D map of the retinal distribution [27]. While this tool offers reliable and reproducible MPOD measurements, it does not provide a detailed spatial analysis offered by 2-D MPOD system, which is essential for a comprehensive understanding of macular pigment distribution in children.

Moreover, serum L and Z levels were not measured. While dietary intake using the KIDMED questionnaire provides an indirect measure of the adherence to a carotenoid-rich diet, it does not reflect actual carotenoid status. Hence, further studies are warranted to clarify this association between serum levels of L and Z, MPOD, and visual function in pediatric populations. Finally, the generalizability of our findings may be limited by the homogenous nature of the sample, which included only healthy White children. Additionally, all participants exhibited medium or high adherence to the Mediterranean diet, which may have precluded the detection of potential differences in visual function associated with low adherence.

## Conclusion

In conclusion, this study found no correlation between visual function parameters –such as refractive status, visual acuity or contrast sensitivity– and MPOD in White children who had medium versus high adherence to the Mediterranean diet. Furthermore, no significant differences were observed in visual function between groups with different levels of Mediterranean diet adherence, and subsequently lutein and zeaxanthin consumption. These findings suggest that, within this population, moderate variations in dietary carotenoid intake may not strongly impact MPOD values or visual performance. Further studies with larger and more diverse samples, including children with low diet adherence and broader refractive error ranges, are needed to clarify these relationships.

## Electronic supplementary material

Below is the link to the electronic supplementary material.

Supplementary Material 1
