# Supplementary material for: “Characterization of visual function parameters in relation to macular pigment optical density in a pediatric population”
Source: Graefes Arch Clin Exp Ophthalmol. 2025 Sep 8;263(12):3529–35. doi: 10.1007/s00417-025-06935-1 (PMC12886255; doi:10.1007/s00417-025-06935-1)
Supplement: Supplementary file 1 — Supplementary Material 1 [file 417_2025_6935_MOESM1_ESM.docx]

**Appendix: KIDMED Questionnaire – Mediterranean Diet Quality Index for children and adolescents.**

(Adapted from Serra-Majem et al.)

| Item | Response | Score |
| --- | --- | --- |
| 1. Takes a piece of fruit or fruit juice every day | YES/NO | +1 |
| 2. Has a second piece of fruit every day | YES/NO | +1 |
| 3. Has fresh or cooked vegetables regularly once a day | YES/NO | +1 |
| 4. Has fresh or cooked vegetables more than once a day | YES/NO | +1 |
| 5. Consumes fish regularly (at least 2–3 times per week) | YES/NO | +1 |
| 6. Goes more than once a week to a fast-food (hamburger) restaurant | YES/NO | -1 |
| 7. Likes pulses and eats them more than once a week | YES/NO | +1 |
| 8. Consumes pasta or rice almost every day (5 or more times per week) | YES/NO | +1 |
| 9. Has cereals or grains (bread, etc.) for breakfast | YES/NO | +1 |
| 10. Consumes nuts regularly (at least 2–3 times per week) | YES/NO | +1 |
| 11. Uses olive oil at home | YES/NO | +1 |
| 12. Skips breakfast | YES/NO | -1 |
| 13. Has a dairy product for breakfast (yoghurt, milk, etc.) | YES/NO | +1 |
| 14. Has commercially baked goods or pastries for breakfast | YES/NO | -1 |
| 15. Takes two yoghurts and/or some cheese (40 g) daily | YES/NO | +1 |
| 16. Takes sweets and candy several times every day | YES/NO | -1 |
